# The Role of Machine Learning and Design of Experiments in the Advancement of Biomaterial and Tissue Engineering Research

**DOI:** 10.3390/bioengineering9100561

**Published:** 2022-10-17

**Authors:** Ghayadah Al-Kharusi, Nicholas J. Dunne, Suzanne Little, Tanya J. Levingstone

**Affiliations:** 1 School of Mechanical and Manufacturing Engineering, Dublin City University, Dublin 9, Ireland; 2 Centre for Medical Engineering Research (MEDeng), Dublin City University, Dublin 9, Ireland; 3 Advanced Processing Technology Research Centre, Dublin City University, Dublin 9, Ireland; 4 Advanced Manufacturing Research Centre (I-Form), Dublin City University, Dublin 9, Ireland; 5 Biodesign Europe, Dublin City University, Dublin 9, Ireland; 6 Trinity Centre for Biomedical Engineering (TCBE), Trinity Biomedical Sciences Institute, Trinity College Dublin, Dublin 2, Ireland; 7 Advanced Materials and Bioengineering Research Centre (AMBER), Royal College of Surgeons in Ireland and Trinity College Dublin, Dublin 2, Ireland; 8 School of Pharmacy, Queen’s University Belfast, 97 Lisburn Road, Belfast BT9 7BL, UK; 9 Insight SFI Research Centre for Data Analytics, Dublin City University, Dublin 9, Ireland

**Keywords:** machine learning, biomaterials, Design of Experiment, tissue engineering, 3d printing

## Abstract

Optimisation of tissue engineering (TE) processes requires models that can identify relationships between the parameters to be optimised and predict structural and performance outcomes from both physical and chemical processes. Currently, Design of Experiments (DoE) methods are commonly used for optimisation purposes in addition to playing an important role in statistical quality control and systematic randomisation for experiment planning. DoE is only used for the analysis and optimisation of quantitative data (i.e., number-based, countable or measurable), while it lacks the suitability for imaging and high dimensional data analysis. Machine learning (ML) offers considerable potential for data analysis, providing a greater flexibility in terms of data that can be used for optimisation and predictions. Its application within the fields of biomaterials and TE has recently been explored. This review presents the different types of DoE methodologies and the appropriate methods that have been used in TE applications. Next, ML algorithms that are widely used for optimisation and predictions are introduced and their advantages and disadvantages are presented. The use of different ML algorithms for TE applications is reviewed, with a particular focus on their use in optimising 3D bioprinting processes for tissue-engineered construct fabrication. Finally, the review discusses the future perspectives and presents the possibility of integrating DoE and ML in one system that would provide opportunities for researchers to achieve greater improvements in the TE field.

## 1. Introduction

Tissue engineering (TE) involves the creation of sophisticated three-dimensional (3D) constructs (i.e., cells incorporated within a scaffold) that aim to mediate the repair of injured or diseased tissue. TE can be defined as the combination of the principles of biomaterials and stem cell transplantation to develop and support endogenous tissue regeneration [1]. Cell transplantation plays a key role in TE and is used for therapeutic strategies to treat various injuries, such as bone fractures and cartilage defects. New methods have been developed that include the direct injection of cells to the affected area, reducing surgical invasiveness and its associated risks [2]. Despite its relatively short history (i.e., 40 years) [3], TE has become a fertile ground for scientific discoveries in both applied and fundamental sciences. There has been a tremendous expansion in the field since its initial goal—to address the shortage of tissue and organ donors by creating replacement tissues, such as cartilage, blood vessels, bone, and skin. More recently, tissue-engineered constructs have been applied as drug delivery systems, disease modelling platforms, and high-throughput screening devices [4,5,6]. Traditionally, one-at-a-time type experiments have been widely applied in the development and optimisation of biomaterials and tissue-engineered constructs. However, this approach is slow, expensive and cannot demonstrate the complex interactions between input variables and associated outputs. This results in the slow and arduous development of new biomaterials and tissue-engineered constructs, which delays their potential clinical translation. 

Over the past decade, a one-at-a-time type experimental approach has been superseded by statistical experiments, e.g., Design of Experiments (DoE), where input variables can be altered simultaneously to obtain the maximum amount of information from a minimum number of experiments. This experimental approach involves a series of systematic tests that aim to find the factors that have the greatest effect on response variables [7]. The application of these statistical approaches enables the development of models that can predict the properties of biomaterials and tissue-engineered constructs, identify the relationships between properties and optimise their structural and performance outcomes with a reduction in experimental iterations, saving time, and reducing the consumption of laboratory resources and the overall cost of product development [8].

**Figure 1:**
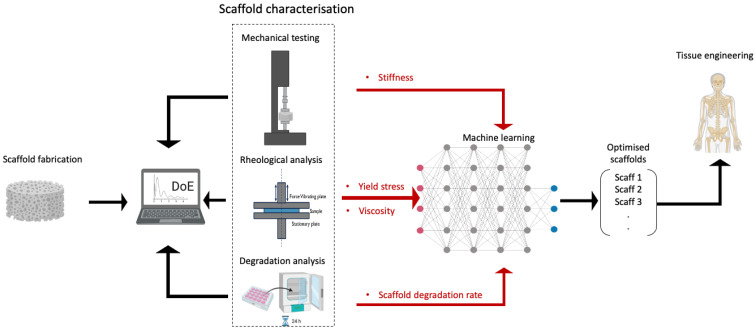
DoE and ML workflow to optimise the material that will be used for the TE applications.

TE generates data from several characterisation techniques, including physicochemical analysis, microstructural analysis, rheological assessment, mechanical testing, and degradation measurements (Figure 1). Although DoE approaches are used in many studies for optimisation problems, these statistical methods may not be compatible for processing and predicting certain types of data, such as images, video, audio and high dimensional data, where the number of features is larger than the number of observations (Figure 1) [9]. Machine learning (ML) has shown the potential to overcome many of these existing experimental challenges, providing new methodologies for optimisation within the field of TE. ML plays a significant role in the world today and its impact is transformational, disrupting society and industry alike. The application of ML has shown the potential to bring about rapid process optimisation [10,11]. Recently, the application of ML in biomaterials and TE research has been demonstrated with the successful use of prediction methods, artificial neural networks (ANN), convolutional neural networks (CNN), Bayesian optimisation (BO) and robot-based rapid prototyping systems, which can be used for prediction and optimisation in TE applications [3,12,13,14,15,16]. Furthermore, ML has been combined with DoE to provide further enhancement of the optimisation process in biomaterials and TE research [17]. Despite evidence highlighting the application of ML reported in the literature, significant challenges remain, in particular relating to effectively handling the preparation and representation of data generated from biomaterials and TE applications. The majority of studies focus on scaffold fabrication processes, such as 3D bioprinting and freeze-drying [9,12,18,19], and scaffold properties [20], with only a few focussing in detail on how ML can be applied and the main outcomes and benefits that can be obtained from using ML methods [12,21,22]. Challenges relating to the implementation of ML in TE applications, largely relate to the limitations in obtaining suitable datasets and the conversion of large datasets into easily accessible and utilisable formats as data is frequently gathered from manual processes [3,10]. Thus, it is crucial to collect and explore a considerable amount of data to extract the right features and make it manageable. As a starting point, this review presents the most widely used DoE methodologies that have evolved into the analytical foundation for more complicated approaches in TE. Following this, ML applications that have been recently used in the biomaterials and TE fields are introduced, illustrating the advantages and drawbacks of their use in these specific fields. Finally, the main differences between DoE and ML methodologies in biomaterials and TE applications are highlighted, and the possibility of combining the two methods to improve the optimisation and prediction process is explored.

## 2. Design of Experiments (DoE)

DoE can be defined as a mathematical approach that is used for planning and performing experiments, data analysis, and interpretation of the conducted experiments. A DoE approach uses a controlled set of tests to model the relationships between factors and observed responses to plan experiments and analyse data. Using this method, researchers can make evidence-based decisions with the minimum number of experiments. Once the effective factors have been identified, DoE methods can be employed to optimise the experimental response variables. To determine the relationship between the factors and the response variables, the DoE variables must be selected carefully, including their ranges and the number of experiments run. DoE can be applied in several types of systems and processes, such as during product design and development, for statistical quality control, to assess the systematic randomisation used for experiment planning, for results of model fitting and optimisation to conduct systematic research of a system [23]. DoE studies should be designed using as few experimental runs as possible when constructing predictive models and making a design or technology decision because each experimental run requires costly and time-consuming experimental analysis [24]. 

**Figure 2:**
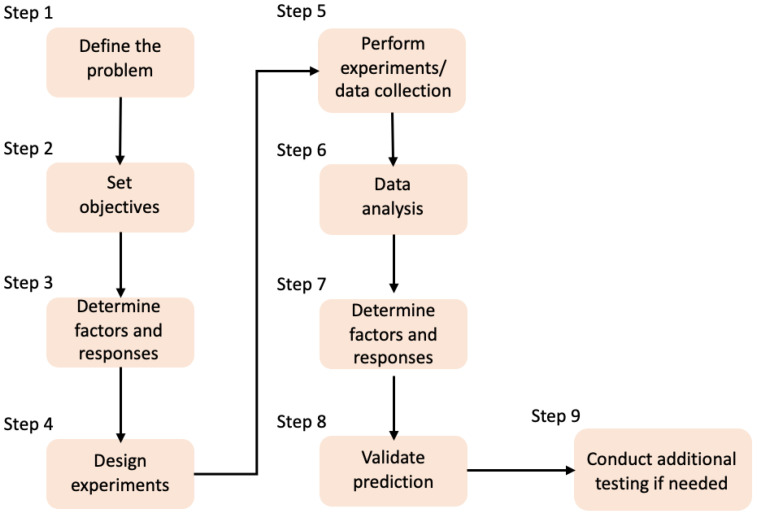
DoE workflow for the process optimisation.

The typical approach of a DoE workflow for process optimisation, is illustrated in Figure 2. In general, the process involves nine steps that can be described as follows: (1) identifying the main project problem, by asking what are the main outcomes of the project; (2) structuring a DoE, which involves planning the experiment and outlining the main objectives of the project; (3) determining the factors, levels, and responses to be investigated in the study, identifying the response assumptions, and defining the methods to be used; (4) the experiment is then completed according to the experimental plan and outputs measured; (5) using output data, mathematical models of the studied process are produced, to meet the study objectives; (6) the built model is evaluated by using the response data and demonstrated visually using plots; (7) the measured responses are then used to identify the significant factors; (8) the final stages of the optimization involve conducting additional experiments to verify the model’s optimal responses; and finally, (9) additional testing is conducted where there is missing data or where further data analysis is required with altered ranges of factors and responses [25]. 

Generally, a DoE approach is applied in a phased way where a screening study is firstly completed. This allows the number of factors to be reduced and the appropriate levels for each factor to be determined before the optimisation. A range of DoE methods has been used for materials sciences and engineering applications, including factorial experiments, Latin squares, Taguchi and response surface methodologies (RSM) [26,27,28]. The following section elaborates on the differences between these methods and how they have been applied to biomaterials and TE applications.

### 2.1. Factorial Experiments

Factorial experiments consist of two or more factors, each with discrete possible levels. For two-level factorial designs, the input factors are set at two levels, a ‘low’ level and a ‘high’ level, defined as ‘−1’ and ‘+1’, respectively. Full factorial designs contain all possible combinations of low and high levels for all input factors. Therefore, if there are k factors, a full factorial design will consist of 2^k^ experimental runs. This type of design is useful when the number of factors to be explored is low. When there are larger numbers of factors, the design becomes inefficient as a large number of experimental runs are necessary e.g., for a two-level design with four factors (2^4^), 16 runs are required, whereas for seven factors (2^7^), 128 runs are required. In these cases, a fractional factorial design can be employed, whereby a fraction i.e., ½ or ¼, etc., of the runs specified in the full factorial design are carried out. For example, a full factorial design with three factors at two levels, will result in 2^3^ = eight experimental runs, whereas a ½ fraction design, written as 2^3−1^, requires four experimental runs. Fractional factorial designs are only appropriate if the expected interactions between the factors are negligible in comparison to the main effects. The factorial designs are best suited for screening experiments completed to select the main effects within an experiment. Three-level factorial designs examine the factors at three levels, ‘low’, ‘intermediate’ and ‘high’ or ’−1’, ‘0’ and ‘1’ [29].Three-level designs enable quadratic responses to be investigated, however, these quickly become prohibitive in terms of the number of runs required.

### 2.2. Latin Square

**Figure 3:**
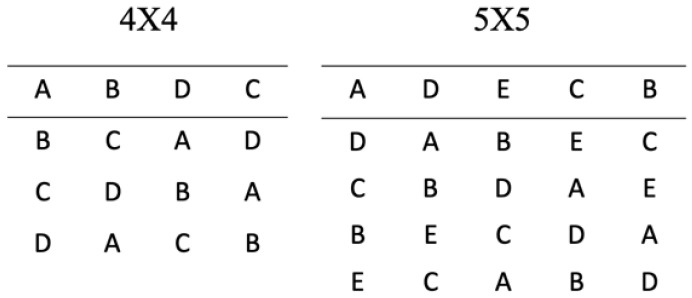
Latin square of each treatment is repeated n times so that it appears once in each row.

A Latin square design consists of an *n* × *n* array filled with *n* different symbols, resulting in a square table of *n*^2^. As shown in Figure 3, a Latin square is a table filled with different Latin letters (A, B, C etc.), that correspond to the treatments. The main rule for the Latin squares is that these symbols can only occur once in each row and column. The number of experimental runs required will equal the number of treatment conditions investigated. Latin square design cells are mainly used to remove two unwanted sources of variability within an experiment. The process involves blocking in two directions. Hence, the rows and columns represent two limitations on randomization. Latin squares are equivalent to specific fractional factorial designs, e.g., a 4 × 4 Latin square design is equivalent to a 4^3−1^ fractional factorial design. A full explanation of the statistical representation of Latin square designs is given by Montgomery et al. [7].

### 2.3. Taguchi Designs

**Figure 4:**
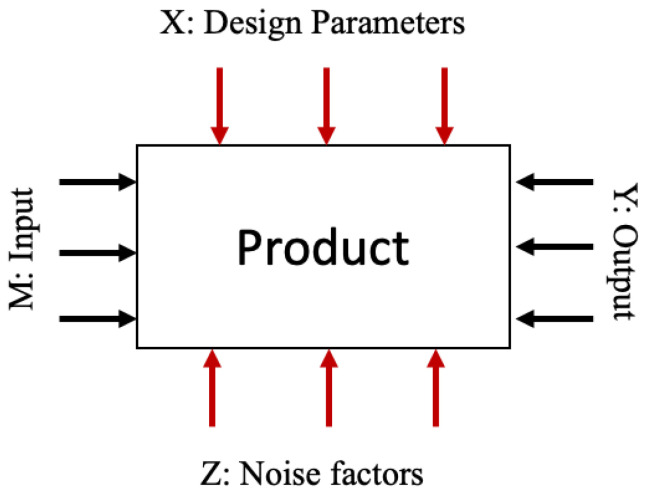
P-diagram. A P-diagram is used to classify the variables associated with the product into input energy, signal or user intent (M), noise factors (Z), design parameters (X) and output/key product characteristics, functions, performance, etc. (Y).

Taguchi designs are types of fractional factorial designs that involve a subset of combinations of multiple factors at multiple levels. The factors are divided into two sets: (1) control factors, which are under our control, and (2) noise factors, which vary due to the experimental environment and are not controlled. The noise factors can vary during the experimental environment even though they have no direct control [30]. Taguchi designs involve the optimisation of a process that has several control factors which directly affect the output target or desired value. These variables can be classified into inputs (M), noise factors (Z), design parameters (X) and outputs (Y), as illustrated in the P-diagram in Figure 4 [31]. The Taguchi design employed will depend on the objectives of the experiment, e.g., two-level Taguchi designs can be used for screening, and other methods can be used for a more detailed investigation of a process [32].

### 2.4. Response Surface Methodology (RSM)

**Figure 5:**
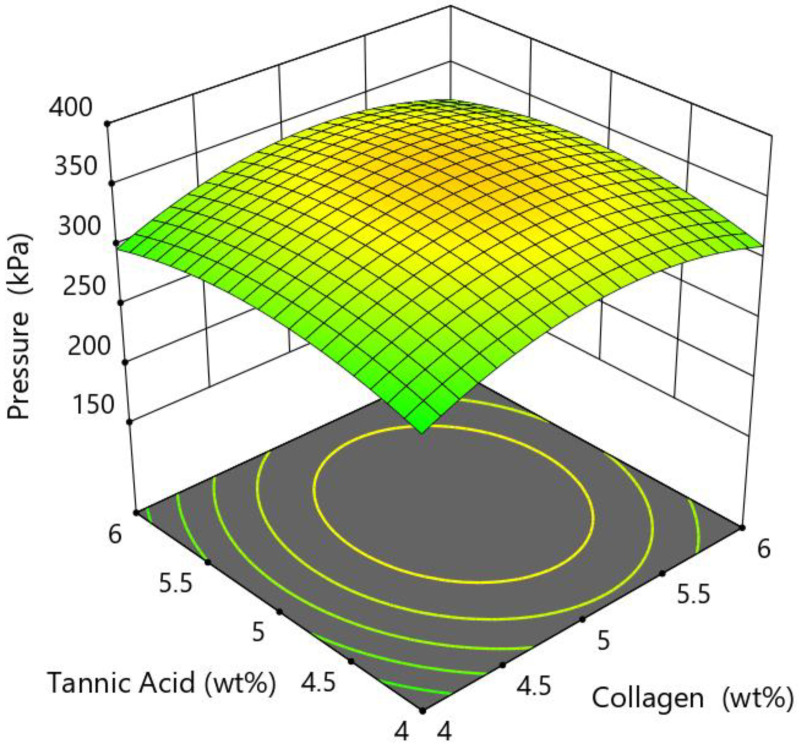
3D response surface example to optimise the 3D bioprinting pressure.

RSM is a collection of statistical and mathematical methods that can be applied in modelling and analysing problems where several input variables affect the response of interest, and the main objective is to optimise this response [7]. The usual representation of the response surface is illustrated in Figure 5. The following example represents two factors—the composition of tannic acid and collagen concentration that influence the printing pressure (response). The relationship between the pressure and these two factors represents the response surface.

**Figure 6:**
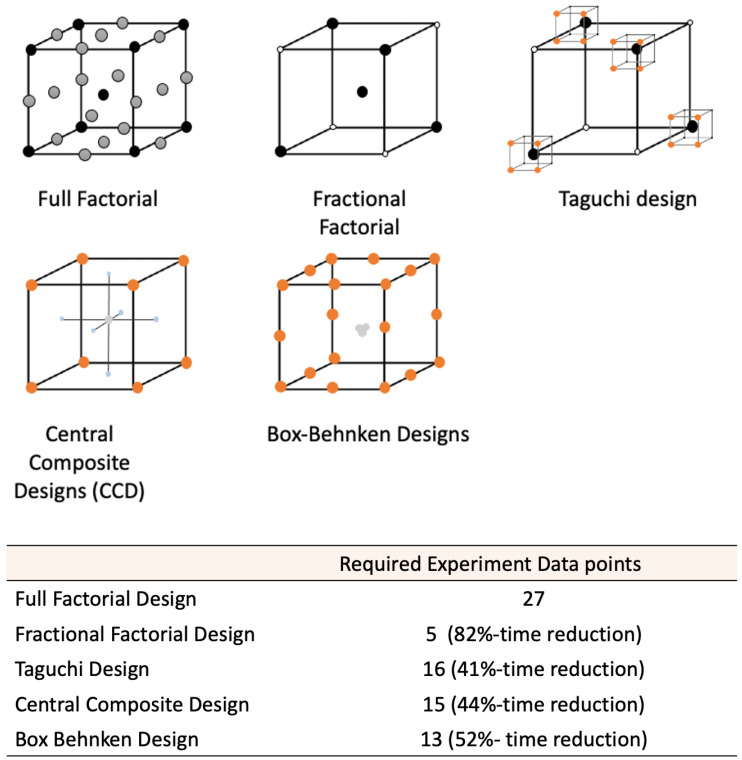
Number of experimental runs required for each design for 3 factors investigated at 3 levels.

The two main groups of RSM designs are (1) central composite designs (CCDs) and (2) Box–Behnken designs (BBDs). Both designs provide an understanding of the behaviour of a system (i.e., reveal the connection between factors and responses) and enable its optimisation. CCDs are usually applied after a process of screening has narrowed down the important factors. It contains central and axial points in addition to cube points, which allow the estimation of higher-order effects, based on a curvature of the response [33]. BBDs have fewer experimental runs than CCDs and do not have points at the vertices of the cube (i.e., low and high points). As a result the prediction quality of BBDs, the quality is lower than the standard CCDs, however, they offer advantages for physical experimentation because extreme points are time-consuming and costly to investigate [33]. RSM is primarily aimed at optimising a system and can also be used to assess interactions and higher-order terms (e.g., quadratic or cubic), which is not feasible with other experimental design methods. The choice of the DoE methodological approach depends on the problem that needs to be investigated and the main experimental objectives [34]. The data points required for each DoE method, are summarised in Figure 6. Table 1 summarises different DoE techniques in terms of their methodology and the benefits for each method. 

### 2.5. Statistical Tools 

In addition to selecting representative runs that successfully sample the domain of research, dedicated procedures for post-processing the experimental results need to be used. These methods yield both qualitative and quantitative data relating to the impact of the many independent variables on the dependent variables. The analysis of variance (ANOVA) method is a mathematical and statistical process for determining whether there are differences between the means of groups within a sample and whether these differences are random or can be related to a particular cause. In DoE, the sample represents a set of experiments completed in accordance with a predetermined plan while groups within a sample are a collection of data connected to a specific factor, level or response. ANOVA breaks down the total variance and allocates it to all the distinct causes by comparing the group means of a sample. As a result, it may be used to quantify the effect of factors and responses on the independent variables [35].

**Table 1:** Overview of the Design of Experiments (DoE) techniques.

Techniques	Overview	Methodology	Benefits	Ref
Factorial designs	All factors are assessed as all possible combinations of ‘high’ and ‘low’ levels. Fractional factorial designs can be used to reduce the number of experimental runs.	Usually involve two or more factors assessed at two levels.	Useful for determining the main effects in screening experiments; Straight-forward to design; Robust.	[29]
Latin square	Ideally used for experiments in which it is possible to test subjects individually under every treatment.	Number of experimental conditions is required to equal the number of different labels	High control of the variation from the different experimental runs and labels Better efficiency compared to other techniques.	[34,36]
Taguchi designs	Determination of the best combination of inputs to produce a design or a product.	Determines parameter levels.	Identifies the right input; High-quality product; Robust design perspective.	[30,37]
Response Surface Methodology (RSM)	An offline optimisation method, which usually involves studying two factors. However, this technique can be used to study three or more factors. The method is usually employed in optimisation experiments.	RSM merges mathematical and statistical methods with experimental designs, to develop models that relate to the response and control factors.	Represents relationship between the responses and control factors; Allows response values to be predicted using a range of control factors; Provides optimum values for control variables; Uses statistical testing to determine a significant control variable.	[37,38]

### 2.6. Comparison of the DoE Techniques

The DoE techniques described have various advantages and disadvantages and the choice of design will depend on the objectives of the experiment and the number of factors that need to be investigated. Two-level factorial experiments are best suited for the investigation of main effects or as screening designs. Latin square and Taguchi designs are also best suited for screening experiments. Taguchi designs are often utilised for very large screening experiments. Three-level factorial designs and RSM techniques are more suited for studying interactions between factors, for process optimisation, troubleshooting process problems and the assessment of the overall robustness of a process. Generally, it is recommended to complete a screening design to determine the main effects before optimisation to reduce the numbers of factors required and to ensure the suitability of the levels selected for each factor. In the selection of the most suitable RSM technique, it is useful to consider the number of experimental runs required for each design. Considering an experimental design, consisting of three factors at three levels, a full factorial design will require 27 experimental runs, a BBD will require 13 experimental runs and a CCD will require 15 experimental runs. Although the CCD approach requires a greater number of experimental runs, it has advantages in that it can include up to five levels per factor and they allow for the inclusion of runs where all factors are at their extreme settings, e.g., all at the high settings.

### 2.7. Application of DoE Methods in Biomaterials and TE Research

There have been many studies applying DoE methods in biomaterials and TE research which include the optimisation scaffold fabrication methods [34,39,40,41,42,43], hydrogels [44], bioactive extraction methods [45], electrospun materials [46], 3D bioprinting. In particular, 3D bioprinting [47], an emerging tissue-engineered construct fabrication technique in TE, has been the focus of a number of studies [48,49,50]. This fabrication technique involves the layer-by-layer deposition of bioinks to produce complex structures designed to generate functional tissue or organs [51,52,53,54]. The process can be divided into three steps: (1) pre-printing, which includes the bioink formulation and pre-processing, (2) printing, where the in-situ printing parameters need to be optimised and corrected, and (3) post-printing, involving the optimisation of the culture conditions to achieve a functional tissue/organ.

DoE has been employed to explore the influence of the bioink properties, printing parameters and scaffold design on the properties of the resultant construct [48,49,50]. For instance, Trachtenberg et al. applied a full-factorial design to investigate the influence of poly (propylene fumarate) (PPF) concentration, printing pressure, printing speed and fibre spacing on the bioink viscosity, fibre diameter and pore size of 3D printed scaffolds [50]. The study generated linear models relating the PPF concentration to the shear-thinning behaviour of the bioink, and fibre-spacing and the pressure to the pore size and fibre diameter. Overall, the work provided statistical models with the potential for evaluating the 3D printing compatibility of novel biomaterials and for optimizing the extrusion of these materials for fabricating 3D scaffolds with predictable architectures.

Bhargav et al. optimised the surface morphology and structure of 3D printed scaffolds using a Taguchi design [48]. This study optimised the scaffold surface morphology by altering the following morphological parameters: (1) pore size, (2) fibre diameter, (3) fibre orientation and (4) the number of layers [48]. A Taguchi design was used to understand the relationship between these input parameters and their effect on the mechanical and morphological properties of the resultant construct. These structural parameters play a key role in cell attachment to the construct. In this study, the construct was designed as a square mesh. The adoption of a Taguchi design reduced the number of experiments required by evaluating each parameter, using an orthogonal array, where four factors (i.e., pore size, fibre diameter, fibre orientation and number of layers) were evaluated at three levels. The results showed the effect of the four factors on the mechanical properties of the construct [48].

RSM has also been applied for the optimisation of tissue-engineered constructs. Shizard et al. investigated the relationship between the architecture and mechanical performance of the constructs fabricated using 3D printing, using the RSM technique [49]. The study showed the influence of the pore size, architecture and porosity, on the mechanical properties of both uniform and gradient constructs designed for the TE applications relating to hard tissue repair. Specifically, the study aimed to simultaneously optimise the physical, mechanical and biological properties of the construct using the CCD method. The study investigated two factors, based on the geometric parameters of the scaffold, i.e., (1) strut length and (2) strut radius. The porosity and Young’s modulus of the construct was determined using the experimental methods and finite element analysis (FEA) modelling. FEA is a widely used computational approach for the analysis of stress distribution within complex geometries and the optimisation of the mechanical properties of a designed element [55]. The use of simulation models, such as the FEA, can be expensive and time-consuming, and requires expensive software and significant processing power, therefore, their utility is limited. Applying DoE methods to simulations allows for the creation of surrogate models that have a sufficient predictive performance and can be utilised to explore the broad domains in a quick and straightforward manner [56,57]. 

BBD has also been applied for the investigation of other TE fabrication techniques. A recent study by Dehghan et al. demonstrated the use of a BBD to determine the mathematical relationship between the input factors and the responses to optimise the constructs fabricated using the electrospinning technique [58]. The study demonstrated the effect of varying the concentration of the three different constituents within a polycaprolactone/gelatine/polydimethylsiloxane (PCL/GEL/PDMS) composite biomaterial with respect to the strength, elongation, biodegradability and toxicity of the resultant electrospun constructs. The study assessed PCL and GEL within the range of 0–100 wt.% and PDMS in the range of 1–30 wt.%. The results from the RSM described the optimal polymer ratio to achieve the optimal mechanical properties, biodegradability and biocompatibility. The study also determined the relationship between the responses, e.g., it showed that the elongation under the mechanical loading and the biocompatibility demonstrated a quadratic relationship [58]. 

Overall, these studies demonstrate the successful use of Taguchi designs and CCD for tissue-engineered construct design and BBD for biomaterial optimisation. While a direct comparison of the DoE methodologies has not been completed in the context of biomaterials and TE development, Jankovic et al. compared different DoE methods applied to the thermal behaviour of a double skin facade to determine the approach that enabled the best characterisation of the process with the fewest experimental runs [57]. This study demonstrated that the RSM CCD showed the best performance, however, the most efficient design that best balanced the number of experimental runs and accuracy was a Taguchi L18 array 2L + 3L × 2F. They report that the extent of the nonlinearity in the process influences the optimal design. If the higher-order terms are significant, some of the RSM designs are advisable. Whereas, if only the main effects and interactions influence the response quantity, then a Taguchi design of a lower resolution is sufficient. Jankovic et al. also highlighted that during the selection of the optimal design, the physical limitations of the experiment, such as time and material resources and the ability to perform experiments under extreme conditions must be carefully considered and the selected design should secure a comprehensive picture of interactions, using as few resources as possible during the physical experiment. Large data sets and certain types of data cause significant challenges for the DoE methodologies. Correlational or ML methods are better suited for the analysis of big data sets [24]. The next section of this review discusses the potential benefits of introducing ML into biomaterials and TE research. 

## 3. Machine Learning (ML) 

ML has the potential for application in a range of biomaterials and TE applications, such as materials development, the optimisation of scaffolds, cells, and drug delivery [59]. While the DoE is a powerful tool for the identification of the relationships between input parameters and the reduction of the requirement for costly and time-consuming experiments, the limitations relating to the application of these techniques in biomaterials and TE research remain. The application of ML techniques to these research fields presents new opportunities to utilise data to better customise TE processes. Harnessing the power of ML has the potential to bring about rapid advances within the fields of biomaterials and TE. ML can utilise larger quantities and a wider variety of data, including experimental parameters, sensor observations and images or scans, to extend the potential for identifying key relationships between the properties of tissue-engineered constructs. 

**Figure 7:**
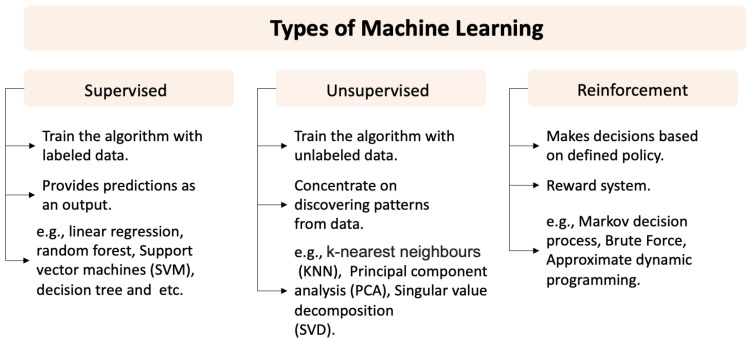
Overview of the ML types that can be used for different applications, supervised, unsupervised and reinforcement learning.

There are different ways to define ML, e.g., according to Alpaydin [60], ML is considered part of artificial intelligence (AI) where the system can learn from given data to produce predictions and to optimise the model parameters using training data. Similarly, Park et al. state that ML can be defined as a field in computer science that can create algorithms that can learn from a big set of data and produces predictions on the data [61]. ML models have shown great improvements in learning complex patterns that enable a model to predict unobserved results and allow computers to train on imported data and use statistical approaches to output results within a certain range [62]. In general, ML techniques can be grouped into three types: (1) supervised [63], (2) unsupervised [64] and (3) reinforcement learning [65], according to how they use labelled data. An overview of these techniques is provided in Figure 7.

### 3.1. Supervised Learning 

Supervised learning is an ML approach that aims to predict unknown outputs using labelled training data, based on prior observations. Supervised learning works by receiving datasets and then training a regression/classification model [66,67,68]. Subsequently, the model can generate predictions to respond to new, unseen input data. There are several types of classification techniques that focus on providing categorisation, data analysis and pattern recognition [68,69]. The benefits and limitations relating to supervised ML techniques that have been previously applied and have potential for greater use in biomaterials and TE applications are summarised in Table 2.

#### 3.1.1. Linear Regression 

Regression analysis can be described as the function used to make predictions on real value outputs, where this function can signify the dependent variable by identifying the independent variables [70]. Linear regression analysis is an approach whereby a linear relationship is modelled to predict or explain the relationship between variables [77]. This linear relationship between two variables can be illustrated as a straight line. A linear regression model, therefore, aims to optimise the fitting of a straight line to a given dataset [69]. However, the use of a straight-line approach can provide an imperfect summary of the complex relationships and can be influenced by randomness in the experimental data.

#### 3.1.2. Decision Tree and Random Forest

A decision tree is a supervised learning algorithm that can be used for classification and regression problems. The approach involves a series of sequential decisions that enable a specific result to be reached. For classification problems, decision trees can be used to segregate a data set into classes that correspond to the response variable. The most basic decision trees involve splitting the response variable into two categories: (1) yes/no or (2) 0/1. Alternative decision tree algorithms can be used if the response variable contains more than two categories. A regression tree is a type of decision tree in which the target variable can take continuous values. They are frequently used to predict numerical problems. The type of decision tree that needs to be applied depends on the desired variable [78]. 

Decision trees are extremely quick and can handle high-dimensional datasets and larger input datasets compared to other approaches [79]. One challenge in applying decision trees is that they can be prone to overfitting issues, where the system tightly fits the training data to the extent that it is inaccurate in predicting the outcomes of new data [80]. While such systems have a very low training error, the test error may be bigger. This problem can be addressed by using random forests, which combine several decision trees, and uses bootstrapping and aggregation to train numerous decision trees, simultaneously [81]. Data and characteristics are randomly selected for each decision tree and an average of the individual decision tree predictions yields the result. Each tree receives data from the original dataset and at each node, a subset of the optional attributes is chosen at random. This approach combines many weak or poorly connected classifiers into one strong classifier [82]. As a result, random forests maintain their speed while also being extremely resistant to overfitting [83]. 

#### 3.1.3. Neural Networks

Neural networks, also known as artificial neural networks (ANNs), are defined as computational models that attempt to imitate the human brain through the use of neuron nodes interconnected in a web. In the human brain, billions of cells called neurons can be found where they are responsible for processing information (i.e., input data) and generating responses (i.e., output data). Similarly, ANNs have hundreds of thousands of artificial neurons, called processing units, as interconnected nodes. More recent models, called deep neural networks or deep learning, have been used extensively for natural language processing and computer vision and can contain billions of neurons. ANNs work by arranging processing units into layers of inputs and outputs. The input layer collects data, then the neural network model uses hidden layers and nodes to learn through many iterative phases and optimise the predicted results as an output [70]. 

Knowledge-based ANNs utilise a hybrid learning approach, combining theoretical knowledge with the knowledge learnt from a set of classified examples, thus enabling them to learn more effectively than the classical ANN approach. In the knowledge-based ANNs, the output of one sub-ANN is used as an input to another sub-ANN in the chain. This allows the hidden layers of the knowledge-based ANNs to work in a more dimensionally uniform environment than the classical ANNs, resulting in a reduction of 12 neurons. This approach has improved the accuracy in training ANNs, while also minimising the prediction error [84]. Nagerejan et al. applied knowledge-based ANNs to develop a metamodel capable of dynamically predicting control factors of a fused deposition modelling additive manufacturing process training time [84]. The study demonstrated the potential of this approach to reduce the dimensionality of the complex additive manufacturing problems.

A further class of ANNs is the convolutional neural networks (CNNs) that apply a convolution function to further refine the outputs. CNNs have been widely used in the material manufacturing sector, mainly using computer vision for detecting defects [32,85,86,87]. CNNs learn using images as input to the model and internal layers that can detect certain features, such as edges and lines in an object. The classifier is trained by using labelled images and can output a class label or labels, localisation of objects or a full segmentation of the image.

#### 3.1.4. Support Vector Machines (SVMs)

A classical and widely used machine learning technique, support vector machines (SVMs) are linear classifiers that predict the class of each input’s members from a set of two possible classes. To classify all inputs in a high-dimensional or even infinite space, SVMs generate hyperplanes or groups of hyperplanes. The closest points of categorization are known as support vectors. SVMs are most concerned with the hyperplane-to-support-vector margin [88]. The success of SVMs in producing accurate findings is due to their ability to train well with only a few features, robustness against model error and computational efficiency when compared to other ML methods, such as ANNs [80]. 

#### 3.1.5. Kernel Ridge Regression (KRR)

A refinement of SVMs, the Kernel ridge regression (KRR), also known as least-square support-vector machines (LS-SVMs), is a non-parametric technique that calculates the target by computing the inputs directly [73]. It is a nonlinear regression method that incorporates regularisation to avoid overfitting. Hyperparameters, i.e., parameters used to control the learning process, and training data size have a significant impact on the performance of the KRR learning model [74]. The approach can be used for classification and regression analysis [89].

#### 3.1.6. Bayesian Optimisation (BO)

Hyperparameters and their optimisation are a key part of machine learning engineering and can have a significant impact on the performance of the model. Bayesian optimisation (BO) is commonly used in ML for hyperparameter optimisation [90] and is very useful in situations where evaluations of a function are costly [91]. Bayesian optimisation is a sequential model-based approach designed to deal with the problem of finding a global minimiser (or maximiser) of an unknown objective function *f*:

$$x^{*}=\arg\underset{x\in X}{\min}f\left(x\right)$$

Where *x* is some design space of interest. Furthermore, the Bayesian optimisation is about maintaining a probabilistic surrogate model over likely functions given in the observed data, and sequentially selecting the future query points according to a selection policy, which leverages the uncertainty in the surrogate to negotiate the exploration of the search space and the exploitation of currently suspected modes [87,88]. This has a particular use for complex scenarios, such as those found in biomaterials and TE, where the model must incorporate the input from several complex systems.

#### 3.1.7. Hierarchical Machine Learning (HML)

One of the major challenges in applying ML to the fields of biomaterials and TE research, is the limited availability of labelled experimental datasets. This can result in highly imbalanced data where there are higher volumes of data from typical or “normal” scenarios and relatively low volumes of input data for the disease, treatment or syndrome cases. Statistical machine learning methods then tend to default to see everything as the typical or majority case. Hierarchical Machine Learning (HML) is used to handle class imbalance with smaller labelled datasets and can be considered a supervised method. HML involves adapting the human learning strategy using multi-level learning [92] and works by compartmentalising and separating the classifications, e.g., between individuals and the diagnosis [93].

### 3.2. Unsupervised and Reinforcement Learning 

Unsupervised learning is the opposite of supervised learning, using unlabelled data to train the model. Unsupervised methods extract unlabelled features from the input data and classify it using self-taught or derived rules. As a result, these models are typically used to uncover hidden or unknown relationships in high volumes of data [64]. Some examples of methods that can be used for unsupervised learning are, the K-nearest-neighbour (KNN) [94], the principal component analysis (PCA) [95] and the singular value decomposition (SVD) [96].

A type of dynamic programming called reinforcement learning uses reward and penalty systems to train algorithms. In this case, the learning system is referred to as an agent, and it learns in an interactive setting. Rewards and penalties are given to the agent, based on how well they complete their assigned tasks. Dynamic programming is used in reinforcement learning to teach an agent how to maximise the reward in a given environment without the assistance of a human. Reinforcement learning has a different purpose than unsupervised learning, which is to identify an action model that maximises the agent’s reward [97] and minimises the risk [98]. There are two types of actions: (1) exploitative and (2) exploratory. Exploitative actions are those that yield the most profit, while exploratory actions are those that have never been attempted before. With the help of these two strategies, the model may gradually learn more about the environment and grasp the inputs that lead to favourable rewards, thus arriving at optimal answers [99]. Some examples of reinforcement algorithms that are frequently used, include the Markov decision process [99], Brute force [100], and dynamic programming [101]. Where biomaterials and TE scenarios produce high volumes of unlabelled case data, these methods can be useful for classification, the clustering of observations, the identification of trends or prediction of contributing inputs.

#### Inductive Logic Programming (ILP)

Inductive logic programming (ILP) is a subfield of ML that uses first-order logic to represent hypotheses and data. Similar to HML, ILP supports the data efficiency regardless of the size, unlike many ML algorithms that have difficulty in generalising from small numbers of training data [102]. ILP provides a number of advantages over other ML methods. ILP systems can acquire knowledge using background knowledge (BK), for example, by utilising a theory of light to comprehend images. It is possible for ILP systems to acquire complicated relational theories, such as cellular automata, event calculus theories, and Petri nets because of the expressivity of logic programming. It is possible for ILP systems to generalise from a single sample, due to the strong inductive bias provided by the BK. Finally, because ILP systems are symbolic, they naturally facilitate lifelong and transfer learning, which is deemed necessary for developing human-like machines [103]. As in other symbolic systems, the main challenge in using ILP is the capture and codification of the knowledge and relationships using first-order logic. These methods reflect a theory of machine learning that with sufficient, codified, expert knowledge, it is possible to build intelligence. Linked data, ontologies and knowledge modelling methodologies have been used in biological, medical and manufacturing domains to capture and apply descriptions of data and relationships.

While many of the ML tools and approaches described here have been successfully applied in TE to target biomaterial development and optimisation, their application still poses many challenges. The following section details how these ML approaches have been previously applied to biomaterials and TE applications. 

### 3.3. Applications of ML in Biomaterials and TE Research

In the years to come, ML will continue to be a crucial part of how science and understanding move forward. As increasingly massive datasets are generated and captured, these technologies have the potential to enhance engineering design and act as more accurate experimental outcome predictors. Numerous other engineering fields have acknowledged the value of these technologies, and have already started to adopt them [101,102]. The development of biomaterials and medical device technologies for TE applications has lagged behind this trend. The use of ML in biomaterials and TE research has the potential to provide researchers with the ability to discover patterns in data, enabling the accelerated development and specialisation of outputs for individualised or personalised solutions. The application of ML in biomaterials and TE is a nascent field and, to date, relatively few studies have explored the use of ML techniques. At this stage, supervised learning methods have been most commonly used in TE. A key requirement for the development of most supervised ML models is the labelled data and even the unsupervised methods that generally require some labelled data to evaluate the performance of the generated models. In TE, these data typically take the form of biomaterial or tissue-engineered construct characteristics, which can be analysed to produce predictions, based on extracted features [13]. Currently, there is a lack of suitable labelled publicly available datasets for the evaluation of ML applications in these. 

**Figure 8:**
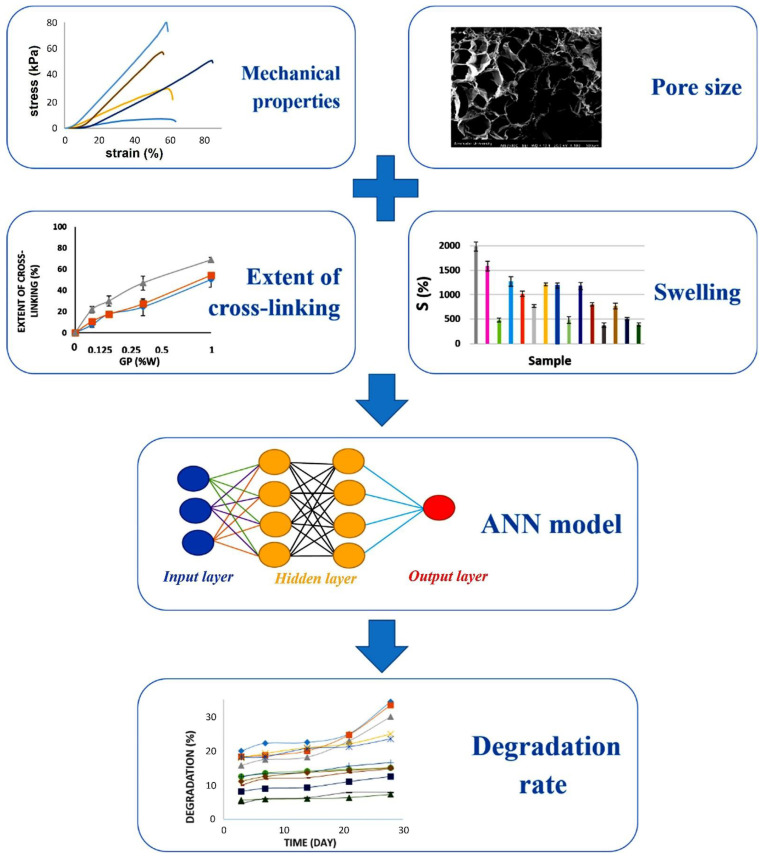
Schematic diagram of the input data (from the material characterisation results) and the output prediction using ML methods. Reprinted with permission from Ref. [18]. 2020, Elsevier.

Recent advances have involved the application of ML for material optimisation, classification and image segmentation [12,18,21,66]. In particular, there has been recent interest in bringing ML approaches to optimise and automate the fabrication processes and provide predictions relating to the material behaviour under certain parameters [9,22,59]. Entekhabi et al. applied the ML approaches to explore the rate of degradation of a freeze-dried gelatin scaffold crosslinked with genipin [18]. In this study, the scaffolds were fabricated using different concentrations of gelatin (2.5%, 5% and 10% (*w*/*v*)) and genipin (0%, 0.125%, 0.25%, 0.5% and 1% (*w*/*w*)) using freeze-drying to create the porous 3D constructs. The rate of construct degradation was measured experimentally by determining the weight change of the scaffold over a 28-day timeframe and collated for mathematical modelling using the data-driven ML approaches. The collected data served as the input for two different supervised learning algorithms, neural networks (i.e., ANN) and KRR. For the purpose of developing an accurate and vigorous prediction, different experimental measurements were selected, according to their correlation with the degradation rate, generating the following variables: porosity, pore size, swelling behaviour, mechanical properties, the extent of crosslinking, and degradation behaviour (Figure 8). The predictions obtained from the different algorithms were compared to the experimental data, showing that ANNs topped the ranking with a mean squared error of 2.68%. Although, in other studies, the KRR has been found to provide a better accuracy than the ANNs, while also having the advantage of not being computationally expensive in training big data [104]. Overall, this study demonstrated the role of ML in saving time and reducing the cost of experimental studies. Further studies can be carried out to explore the different parameters that could be optimised to improve the degradation rate prediction, as well as various other types of data [18]. 

ML has also been recently applied to optimise the various steps of the 3D bioprinting process for the fabrication of tissue-engineered constructs [12,13,14,15,16]. Lee et al. developed a ML-based method for the design of 3D-printable bioinks composed of naturally derived biomaterials. In this study, the relationships between the rheological properties and printability were analysed using ML. The analysis process employed the relative least general generalisation algorithm, an ILP methodology that is useful for classification problems. A multiple regression was used to support the ML results and the prediction of the printability by the ink composition. In this study, 19 samples were used for modelling and six samples for the validation of the prediction algorithm. The study demonstrated a universal relationship between the mechanical properties of the bioink and the printability, showing that a high elastic modulus improves the shape fidelity and extrusion is possible below the critical yield stress. Based on this relationship, various formulations of naturally derived bioinks that provide high shape fidelity were derived using the multiple regression analysis [16].

**Figure 9:**
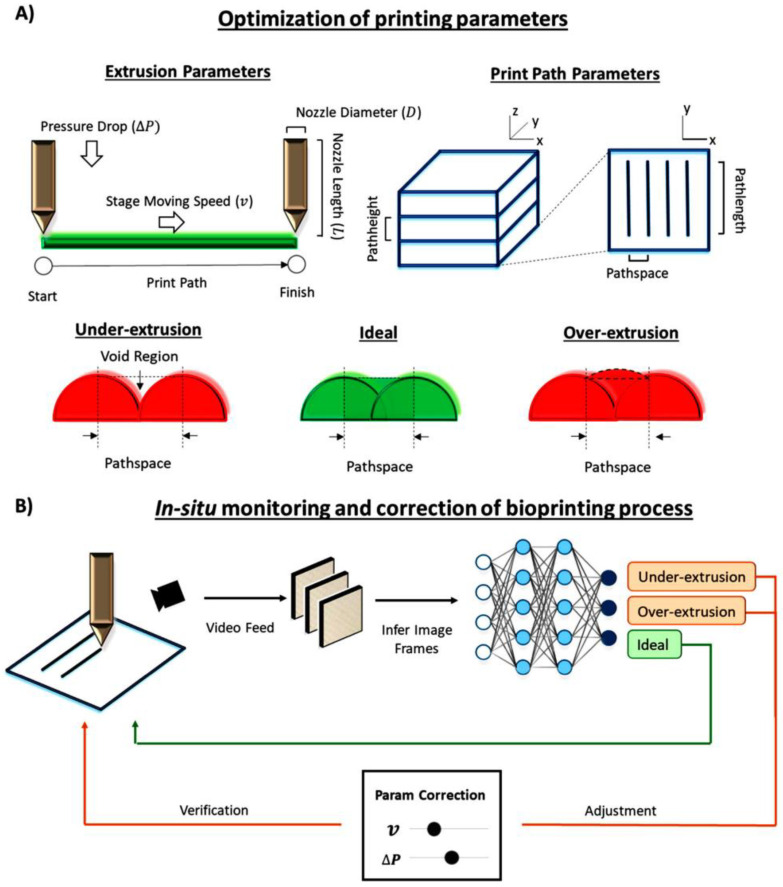
(**A**) Three-dimensional bioprinting optimisation processes. (**B**) Computer vision method used for the optimisation and correction of 3D bioprinting using ML approaches. Reprinted with permission from Ref. [12]. 2020, Taylor & Francis Ltd., www.tandfonline.com (accessed on 12 September 2022).

A recent study by Ng et al. highlighted the potential of applying computer vision and ML to optimise three different bioprinting methods: (1) extrusion, (2) jetting-inject and (3) vat polymerisation–stereolithography [12]. Within their study, they propose mathematical equations that can be used to identify the correlations between the bioprinting parameters for each process [12]. Each bioprinting process has different parameters of interest and different bioinks require unique bioprinting parameters depending on the material behaviour. For the extrusion-based bioprinting techniques, the important parameters that could be investigated using ML techniques, include the printing resolution, the nozzle diameter, the material viscosity, the nozzle length, the stage speed and the change in pressure (Figure 9A). The relationships between these parameters can be modelled using deep learning that can be trained by taking the main printing parameters as inputs and error minimising between the predicted and actual outcomes. By selecting the optimal parameters of the key outputs, e.g., printing resolution, cell viability and fabrication, speed may be influenced [12]. Furthermore, Ng et al. recommended the use of a reinforcement learning agent to automatically select the values for the pressure drop and plate speed. This will help the reinforcement learning to learn and select the ideal values for printing parameter variables that will remove the parameter selections that could result in poor outcomes, such as a low cell viability. This will enable the production of improved bioprinted scaffolds with the desired fabrication speed, higher cell viability and better printing resolution [12]. Finally, the use of in-situ monitoring in bioprinting can help reduce possible errors, such as excess or missing layers of material, to guarantee consistency in the fabrication of 3D bioprinted constructs (Figure 9B). In this study, a CNN classifier was trained by using images labelled as ‘under extrusion’, ‘good quality’ and ‘over extrusion’ (Figure 9B). The schematic diagram represents a feedback loop that adjusts the 3D printing parameters, such as the material flow rate. This is accomplished by using real-time in-situ monitoring (recording images) and the CNN model. This ML approach enables the production of constructs with a higher repeatability and accuracy. This approach can also be used to predict the material properties of a diverse range of bioink compositions and for the development of novel scaffold designs for specific purposes by learning from a huge database of materials and designs [13]. 

Conev et al. have also applied ML for the optimisation of the 3D printing parameters. The study aims to distinguish between low-quality and high-quality printing configurations as a first step toward developing a recommendation system for identifying the optimal printing circumstances. The ML-based framework takes the composition of the material and the printing parameters as inputs and predicts whether the quality of the print will be “low” or “high”. They apply two ML-based strategies: (1) a direct classification-based method that uses a regression ML model to approximatively predict the values of a printing quality metric and (2) an indirect approach that uses an ML model to train a classifier to distinguish between “low” and “high” quality prints. The random forests method is the foundation of both models. One of the main issues faced within this study was the lack of data. Their analysis has revealed that a complete factorial design for data collecting can result in data redundancy in the context of ML [66]. 

One challenge in the application of ML to biomaterial and TE applications is the limited data available for the model generation. Bone et al. addressed the problem of a small dataset by constructing a hierarchal ML (HML) algorithm, wherein the structure of the middle layer leverages the known physical relationships relating to the alginate’s gelation process [15]. This approach was applied to optimise the alginate hydrogel scaffolds fabricated by 3D bioprinting, using the freeform reversible embedding of the suspended hydrogel (FRESH) method. Within the study, the material selection, material formulation and the process parameters were explored to achieve the optimal print fidelity of the printed scaffolds, in terms of linewidth and shape fidelity. The process firstly involved generating a dataset of both high- and low-fidelity alginate prints by systematically varying the print input parameters and assessing the resulting prints in terms of dimensional similarity to the original CAD designs. The model fit was assessed by cross-validation and then optimised to minimize the print error, generating a new set of optimised input variables predicted to generate the high-fidelity prints with an error of less than 10% in dimensionality from the original CAD specification.

An alternative approach to overcome the challenge relating to the small dataset was employed by Ruberu et al., where they utilised the Bayesian optimisation (BO), a sample efficient optimisation algorithm, for the optimisation of the 3D bioprinting of gelatin methacryloyl (GelMA) and hyaluronic acid methacrylate (HAMA) bioinks [14]. The performance of two fundamental criteria encountered in the printing process: (1) the filament formation of the bioink and (2) the layer stacking of the 3D scaffold, were incorporated into a scoring system established to assess the printability. The process involved adding the bioink concentration and printer parameter settings to the optimiser search space and the output recommendations predicted by a ‘black-box’ model were provided to the experimenter where they were scored, based on a visual assessment of the filament morphology and pore architecture.

For numerical simulations, data-driven methods using ML have also been shown to outperform traditional methods grounded in mathematics and physics. Koeppe et al. applied neural networks and deep learning to forecast the stress in a 3D printed lattice structure [19]. The approach involved manufacturing and mechanically testing the lattice specimens. The experimental results were used to validate a parameterised FEA model designed to calculate the stresses in the structures with different design parameters during deformation. Finally, these deformations and design parameters were used to train a neural network. They reported that an ML model takes roughly 0.47 s to predict the stresses, instead of 5–10 h for an FEA simulation [19]. Similarly, Khadilkar et al. used the data-driven CNN to make stress predictions in milliseconds, compared to an FEA method which took a 2–3 min to give stress predictions [105]. 

## 4. Classical ML Techniques Compared with DoE Methods 

The selection of a ML approach over a DoE approach requires a clear understanding of the differences between the two methodologies. Nowadays, DoE is used widely to help optimise processes by reducing the running time and cost of experiments [106]. The process is a human-centred method using a relatively small volume of data, where the researcher is required to select the necessary input factors that need to be included within an experiment, depending on their existing knowledge of the process. By comparison, ML is an automated process, where the data patterns are detected, based on both the input and the output data [107]. According to Freiesleben et al., ML supervised algorithms may be slightly human biased because of the data labelling compared to the unsupervised algorithms but still give better and faster results once trained [106]. In some cases, dealing with a high volume of data, i.e., “big data”, or data with a high dimensionality (p >> n) can have a great effect on the performance of the statistical methods. Typically DoE methods may encounter problems when data are not in numerical format [108], whereas some ML approaches can incorporate different input and output data types. Therefore, the size and type of the data to be used can have a greater influence on the performance of the DoE studies, compared to ML-based studies. 

Comparing the two approaches for application in biomaterials and TE research, it is noted that the use of the DoE methodologies is more effective in terms of experiment reduction, by detecting the most relevant factors, while ML can be used for the high accuracy prediction or classification on a large amount of data. In terms of TE, ML can play an important role in simplifying the modelling of complex interactions involved in multiple biological, chemical, and physical processes in TE. Understanding and defining the principles underlying these processes is considered highly challenging. While standard statistical optimisation, e.g., DoE, has been used in numerous studies in recent years to produce optimal design/fabrication parameters [18], ML has, more recently, shown the potential to produce prediction in terms of scaffold fabrication. The main objective for both ML and the more specific statistical optimisation is to use data to learn and develop mathematical models. The main differences are that statistical optimisation requires a connection between the selected variables to make predictions for the new variables. However, ML can predict data without having to make a new assumption about the actual relationship between the variables. This means that ML treats algorithms, such as a black box [18]. In summary, the two methodologies have two different aims; a DoE primarily focusses on identifying the optimal input factors for the relevant process while ML focuses on identifying patterns in unstructured raw data [106]. 

**Figure 10:**
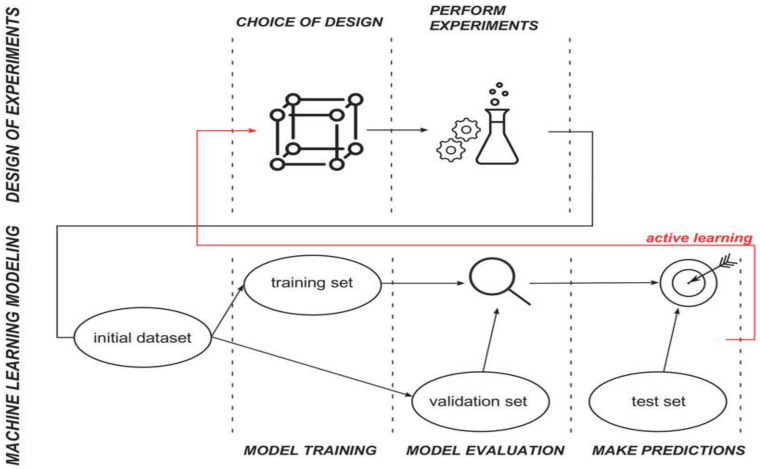
Summarised workflow, describing the link between ML and the DoE, where the DoE data is being used as an input for the ML model. Active learning can occur when the ML result is used as an input suggestion to design a new experiment. Reprinted with permission from Ref. [108]. 2021, John Wiley and Sons.

One challenge in developing ML models is that using large datasets to train a model can be time consuming and expensive. Therefore, the combination of DoE methods with ML holds the potential to further enhance the optimisation within the field of TE. This approach has been successfully applied in product innovation [108], and the chemical [109] and energy consumption industries [110]. DoE data has been used previously in ML algorithms to optimise the initial parameter settings (Figure 10) [106]. In addition, the use of ML has also helped the aim of DoE by detecting the optimal factors and interactions (Figure 10), where the final ML algorithm proposes the next experimental configuration. Therefore, this strategy is often referred to as “active learning” [111] since it puts the learner in control of the data and from that, the machine learns [105,109,112]. 

**Figure 11:**
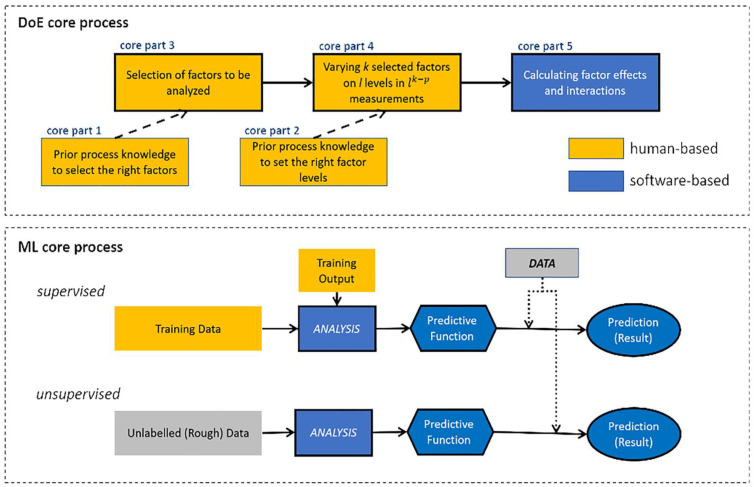
Core processes for the Design of DoE and ML, including human and software-based parts. Reprinted with permission from Ref. [106]. 2019, John Wiley and Sons.

Figure 11 illustrates the core processes for the two methodologies—emphasising the human-based and the software-based parts. This schematic shows how the potential support can be provided to the DoE core process by using ML, and the possibility of replacing the human-based part with a fully software-based core [106]. While DoE and ML are often regarded as if they are independent, in reality, the quality of final results is dependent on both. The initial DoE design decision must be made with the ML algorithm in mind and the ML models should be picked based on the unique characteristics of the dataset collected by the DoE. However, to date, the combined application of DoE and ML approaches has yet to be used to its full potential.

## 5. Summary and Future Perspectives

The present work in biomaterials and TE research has great potential to be improved by applying DoE and ML techniques for material and process development and optimisation. Due to the simplicity of the DoE approach and its advantages over one-at-a-time experimentation, it is widely used in materials and process optimisation and there is significant scope to apply these methods more widely within the fields of biomaterials and TE. However, the approach has limitations relating to the amount and type of data that can be utilised. ML can handle a much higher volume of data with different formats rapidly and consistently. It has the potential for the widespread application in all stages of the ‘bench-to-bedside’ development of tissue-engineered constructs, particularly in the application of image analysis and the phenotypic recognition algorithms, potentially leading to the improved assay or data analysis protocols [113,114]. 

To date, ML has not been widely implemented for biomaterials and TE applications. Numerous challenges prevent the widespread adoption of these techniques within these fields. These include difficulties in obtaining suitable datasets for the development and training of ML algorithms. Currently, data collection is not standardised across different research groups and laboratories, making it difficult to combine/compare data. The development of a big database, necessary for the operation of ML algorithms, relies on the sharing of data. The development and widespread adoption of standards for materials testing, data collecting, and pre-processing would enable more widespread data sharing and stimulate collaboration across the TE field as more groups of researchers work on new materials and processes. A greater integration between ML and TE researchers would also aid in accelerating research within these converging fields, ensuring that complete and labelled datasets in the required formats can be obtained. Furthermore, making data and code publicly available would help the growth and development of the field.

It is difficult to recommend a single specific ML or DoE technique in TE as it depends on the required outputs, i.e., is the research objective to classify, cluster, predict or optimise? In addition, the complexity of the problem under investigation, the resources available and the types of data produced (e.g., literature, experimental, computer experiments, simulation or synthetic data) all need to be considered. The summary information highlighting the key characteristics and applications for DoE (Table 1) and ML (Table 2) may be helpful in identifying the most promising techniques. In addition, for many scenarios, a combination of approaches (sometimes called a ‘polyglot’ solution) will be necessary.

There is also significant scope for the development of new ML techniques designed specifically for application in biomaterials and TE research. Additionally, combining the DoE approach with the implementation of ML models has the potential for enhancing biomaterials and TE research. More research in this area is urgently needed to determine how to best integrate these two methods and explore their application for the optimisation of the bioprinting process, bioink formulations and of other biomaterials and scaffold fabrication processes. Such fusion opens an exciting opportunity for future biomaterials and TE progress. Overall, applying ML techniques within all stages of the development and clinical application of biomaterials and tissue-engineered constructs present exciting new challenges for researchers in both ML and TE and the potential to bring about rapid clinical advancements and improved patient outcomes.

## Figures and Tables

**Table 2:** ML approaches that can be used in Biomaterials and TE applications.

Algorithms	Category	Assumptions	Benefits	Limitations	Ref
Linear regression	Regression	Linearity, fixed features, independence, normality; Error variance is assumed to be constant.	Simple application; Guaranteed to find the optimal solution.	Only works for linear relationship data.	[69,70]
Random forest	Classification	Assume model errors are uncorrelated and uniform.	Provides fast learning and highly accurate predictions; Can intake large set of data without variable deletion; Can work with unbalanced data sets.	Time-consuming to form predictions.	[71,72]
Decision tree	Classification, Regression	The classes must be mutually exclusive.	Easy to use and to understand, efficient algorithm (especially for predictions).	Depending on the selection order, missing factors from the characteristic overfitting.	[71]
Neural networks	Classification, Regression	Variable independence, linearity.	Can be used for classification and regression, able to use the Boolean functions; Allows inputs with noise.	Overfitting due to too many attributes; Hard to understand the algorithm structure.	[71]
Support vector machines (SVM)	Classification, Regression	Model assumptions depend on the probability of default (PD).	Complexity of the model can be easily controlled; The models use non-linear boundaries.	Hard to understand the algorithm structure; Data training is slow.	[69,71]
Kernel ridge regression (KRR)	Regression	Linear or nonlinear function.	Computational simplicity; Prevents overfitting.	Computationally expensive.	[73,74]
Bayesian optimisation (OP)	Optimisation	A non-convex problem; No access to derivative.	Hyperparameter tuning; Cost-efficient.	The objective function can’t be modelled; High dimension problem.	[75,76]

## Data Availability

Not applicable.
